# Supplementary material for: Exploring traumatic childbirth: Associations between obstetric violence indicators and perinatal posttraumatic stress
Source: PLoS One. 2025 Jun 3;20(6):e0324461. doi: 10.1371/journal.pone.0324461 (PMC12132949; doi:10.1371/journal.pone.0324461)
Supplement: Attachment — Submitted filename: Response_Reviewers.pdf [file pone.0324461.s001.pdf]

COMILLAS
UNIVERSIDAD PONTIFICIA
ICAI ICADE CIHS

Madrid, July 18, 2023
Opinion 45/22-23
To: Honorable Vice-Rector for Research and Faculty
Subject: Ethics Committee Opinion on the project titled: "EFFECTS OF OBSTETRIC VIOLENCE ON MATERNAL MENTAL HEALTH AND PARENTING PRACTICES" presented by Professor Amaia Halty Barrutieta from the Family Institute.

The Ethics Committee of the Pontifical University Comillas, following the established procedure and having evaluated the case by its members, issues the following OPINION:

The project safeguards the dignity of the participants. It is a study with clear and socially justified objectives. The participants in the research are first-time mothers over 25 years old who are between four and six weeks postpartum. The participants will gain awareness of the social events that may affect them in the upbringing of their newborn child. The research poses no risks, and all questions asked to the participants are relevant. Therefore, the research project complies with the principles of the Declaration of Helsinki as its benefits outweigh its risks.

The participants in the project have the autonomy to decide whether to participate and are also informed that they can withdraw their consent to participate at any time without needing to provide any explanation. The researcher commits to maintaining confidentiality and anonymity in the data collection so that the identification of participants is not possible. The data derived from the research will be kept at the premises of the Pontifical University Comillas. The research complies with Law 3/2018 on Personal Data Protection and other related legislation.

The project deserves an ethical conformity judgment for research of its characteristics and has the approval of this Committee.

Sincerely,
Dr. Miguel Grande Yáñez President
Dr. Raúl González Fabre Secretary

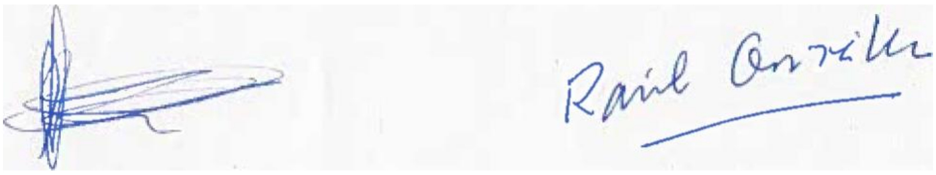

Dr. Miguel Grande Yáñez

Dr. Raúl González Fabre

UNIVERSIDAD PONTIFICIA COMILLAS
Alberto Aguilera, 23. 28015 Madrid Tel.; (+34) 91 542 28 00 www.comillas.edu

Wednesday, November 30, 2022

ETHICAL APPROVAL OF MASTER'S THESIS PROJECTS FROM THE MPGS OF PONTIFICAL COMILLAS UNIVERSITY

Dear master’s Thesis Student,

From the coordination of master’s Thesis Projects of the master’s in general health psychology at Pontifical Comillas University, with the approval of the Master's direction, we are pleased to inform you that your Master's Thesis project titled:

"*Impact of obstetric violence on post-traumatic stress and the quality of the mother-child bond*."

has received approval to carry out the proposed study during the 2022/23 academic year. The code associated with this approval is as follows:

MPGS_2022_008

Best regards,

María Cantero García
Coordinator of Master's Thesis Projects of the MPGS
Pontifical Comillas University

June 4 th, 2024

Response to Reviewers

PONE-D-24-22093

"Based on our evaluation of your manuscript and the information you provided, we do not feel that your submission meets our ethical requirements for human subject's research submissions. PLOS ONE requires that research meets all applicable standards for the ethics of experimentation and research integrity (http://journals.plos.org/plosone/s/human-subjects-research)."

"The ethics approval document you have provided does not meet our standards for approval documentation. It is therefore not clear whether you obtained the necessary ethical approval for the study to take place. As a result of these concerns, we cannot consider the manuscript for publication at PLOS ONE."

Dear Editor,

We appreciate the time and effort you have taken to review our manuscript and the accompanying documentation provided with our submission. We understand and respect your authority to reject any submission that does not meet your internal ethical standards. However, we have resubmitted this paper as we ask you to reconsider your approval of the revision of this manuscript. We believe that there may have been a misunderstanding caused by the documentation we initially submitted.

Our work has been evaluated twice, once by the Institutional Review Board and once by the Ethics Committee of our university, according to its section "Research involving human subjects". This was necessary because the project was carried out in two phases. As detailed in the attached documents. The first part of this work was conducted as a postgraduate thesis and received approval from the Institutional Review Board of the Universidad Pontificia Comillas, Madrid, Spain. Subsequently, as we decided to continue with this line of research, the project was reevaluated and approved by the University's Ethics Committee at the Universidad Pontificia Comillas, Madrid, Spain.

As you can see (as well as in the documents attached as annexes), we have obtained ethical approval to conduct the study on two occasions, as required by its "Human Subjects Research" section. We understand that the document we initially submitted corresponds to the first phase of the study and that this may have caused some confusion.

We have reviewed the guidelines provided at: http://journals.plos.org/plosone/s/human-subjects-research and we believe that this submission meets all of them:

- We obtained prior approval for the research involving human subjects from an Institutional Review Board (IRB), followed by an Ethics Committee.
- We have provided documentation from both the IRB and the Ethics Committee confirming the approval of the research.
- We declared compliance with ethical practices when submitting the manuscript.
- We explained in the manuscript how informed consent was obtained for the research.
- The manuscript and the ethics committee approval document state that the clinical investigation was conducted following the principles expressed in the Declaration of Helsinki.
- No identifying information of the participants has been submitted or provided.

We respectfully request that, considering the possible error on our part in relation to the documentation provided, you may re-appraise this manuscript for publication in PLOS ONE. We continue to believe that our research aligns with and extends the existing body of work in the journal and may be of great interest to readers in advancing scientific understanding of maternal mental health and its influence on postpartum outcomes.

Thank you for your understanding and consideration.

Human Participants Research Checklist

Complete the following if your study involved human participants or human participants' data. These questions should be addressed for prospective and retrospective studies.

1. Did you obtain ethics approval for this study?

    - If yes, please upload (file type "Other") the original approval document you received from your ethics committee. If the original document is in another language, please also provide an English translation.

      _X__ Uploaded ___ N/A

    - If you did not obtain ethical approval, please explain why this was not required below.

2. If you prospectively recruited human participants for the study – for example, you conducted a clinical trial, distributed questionnaires, or obtained tissues, data or samples for the purposes of this study, please report in the Methods:

    i. the day, month and year of the **start and end** of the recruitment period for this study.

    ii. whether participants provided informed consent, and if so, what type was obtained (for instance, written or verbal, and if verbal, how it was documented and witnessed). If your study included minors, state whether you obtained consent from parents or guardians. If the need for consent was waived by the ethics committee, please include this information.

    __X_ Completed ___ N/A

3. If you are reporting a retrospective study of medical records or archived samples, please report in the Methods section:

    i. the day, month and year when the data were accessed for research purposes

    ii. whether authors had access to information that could identify individual participants during or after data collection

    ___ Completed __X_ N/A
