# Supplementary material for: Exploring traumatic childbirth: Associations between obstetric violence indicators and perinatal posttraumatic stress
Source: PLoS One. 2025 Jun 3;20(6):e0324461. doi: 10.1371/journal.pone.0324461 (PMC12132949; doi:10.1371/journal.pone.0324461)
Supplement: Attachment — Submitted filename: Rewiever comments.docx [file pone.0324461.s002.docx]

| **1º Reviewer(s)' Comments to Author** | **Manuscript in general** |
| --- | --- |
| The concept of Perinatal post-traumatic stress symptoms (P-PTSS) is not defined. Considering the wide amount of research about the topic that can be found on PubMed, some readers may misunderstand the concept and confound it with Postpartum post-traumatic stress symptoms or even disorder. This must be clarified. Defining also perinatal period can help in this matter. | Thank you very much for your suggestion. We have clarified what is meant by the perinatal period and completed the definition of perinatal post-traumatic stress at the beginning of the manuscript. Please find attached the paragraph as it would appear after the changes:  *“Childbirth can be experienced by women as a traumatic event that may result in perinatal post‐traumatic stress symptoms (P-PTSS). It is important to note that the perinatal period encompasses not only childbirth but also pregnancy and the immediate postpartum phase. Following the recognition that some experiences during pregnancy and childbirth are sufficiently traumatic to lead to clinical conditions of Post Traumatic Stress Disorder (i.e., PTSD) (1), the term “Perinatal Post Traumatic Stress Disorder” (P-PTSD) was introduced to describe these clinical conditions. P-PTSS refers to the development of posttraumatic symptoms derived from or related to events occurring throughout the perinatal period, and it is distinct from postpartum PTSD or symptoms that arise solely after childbirth. These symptoms often include feelings of fear, terror, hopelessness, or helplessness (1-5).”* |
| The concept of OV is presented first in a simple way, but after, the authors develop a complex definition and I think the first paragraph of the sub-theme should be deleted and the create a new introductory paragraph considering all the concept dimensions. | Thank you very much for your suggestion. We have rewritten the introduction to provide a more integrated definition of OV, addressing the key dimensions of this construct.  *“Obstetric Violence (OV) encompasses a range of practices during childbirth that violate women's autonomy, dignity, and rights. It can manifest physically through medical interventions performed without consent or with insufficient information, and psychologically, through degrading or infantilizing treatment by healthcare providers (15). These experiences can lead to feelings of vulnerability, guilt, and loss of control, impacting maternal well-being and birth outcomes (3,5,16).*  *Physically, OV includes procedures carried out without proper consent or under explicit refusal, such as episiotomies, repeated vaginal examinations by different professionals, the Kristeller maneuver, medically unnecessary acceleration or inhibition of labor, and the forced adoption of specific birthing positions without clinical justification (16). The WHO does not recommend these practices due to their potential negative consequences, including chronic pain, dyspareunia, perineal trauma, and urinary or fecal incontinence (17).*  *Psychologically, OV occurs when women are subjected to dehumanizing, dismissive, or authoritarian behavior by healthcare personnel. This includes being ignored, treated with sarcasm, denied the right to express emotions, or made to feel guilty for childbirth (3,5,16). In this context, the psycho‐affective needs of women—such as emotional validation, empathetic communication, and a supportive environment—are severely compromised. This neglect intensifies feelings of vulnerability, loss of agency, and isolation, thereby exacerbating the adverse psychological effects and increasing the risk of developing perinatal post‐traumatic stress symptoms (P-PTSS). Additionally, a lack of respect for the woman's birth plan, as well as coercion in decision-making, further contributes to these lasting emotional consequences (18)”* |
| Also, I think this part of the paper will benefit from some restructuring as is a little bit "messy" when reading it, some lines are a collection of factors and are hard to read without losing the train of thought. | We sincerely appreciate the reviewer’s insightful comments, which have helped us improve the clarity and structure of our introduction.  We have added subtitles within the introduction to better organize the information and ensure a more logical flow of ideas. Additionally, we have restructured several paragraphs, dividing complex ideas into more readable sections to enhance clarity and readability. |
| In addition, at least one contrasted dimension not mentioned in this manuscript: psycho-affective needs to be addressed. | Thank you for your suggestion. We have expanded on the psychological dimension affected by the presence of OV by specifically highlighting its psycho-affective aspect. Please find attached the paragraph referring to this issue:  *“Psychologically, OV occurs when women are subjected to dehumanizing, dismissive, or authoritarian behavior by healthcare personnel. This includes being ignored, treated with sarcasm, denied the right to express emotions, or made to feel guilty for childbirth (3,5,16). In this context, the psycho‐affective needs of women—such as emotional validation, empathetic communication, and a supportive environment—are severely compromised. This neglect intensifies feelings of vulnerability, loss of agency, and isolation, thereby exacerbating the adverse psychological effects and increasing the risk of developing perinatal post‐traumatic stress symptoms (P-PTSS). Additionally, a lack of respect for the woman's birth plan, as well as coercion in decision-making, further contributes to these lasting emotional consequences (18)”* |
| 3rd. I´ve got problems finding the true aim or goal of the study. There are a few lines where the authors point out the aim pursued, but some of them are just either ambiguous or provoke some degree of contradiction. E.g.: Lines 69-70 VS Lines 140-141. In fact in some parts of the manuscript seems like we are addressing P-PTSS as a result of OV, but in some others feels the other way round. Some readers may fall into the bidirectionality of the phenomena. | We appreciate your feedback and have carefully reviewed the introduction, clarifying the terminology and ensuring that the study’s objective is clear and consistent. We eliminate ambiguities regarding the directionality between OV and P-PTSS, ensuring coherence throughout the text. |
| Methods: Please add in table one also net numbers apart from %. | Thank you very much for the invitation. We have included in Table 1, a new column (N) in which we have included the absolute value of each subject presenting each of the variables listed in the table. It would be as follows: Table 1. General aspects of childbirth. \| Total Sample (N=236) \| \| \| \|; Variable \| N \| % \| \| \|; Complications during pregnancy \| \| \| \| \|; Yes \| 62 \| 26.3 \| \| \|; Type of delivery \| \| \| \| \|; Natural \| 183 \| 77.5 \| \| \|; Scheduled cesarean section \| 7 \| 3 \| \| \|; Emergency caesarean section \| 46 \| 19.5 \| \| \|; Complications during delivery \| \| \| \| \|; Yes \| 53 \| 22.5 \| \| \|; Baby's gestational week at birth \| \| \| \| \|; Extremely preterm \| 1 \| 0.4 \| \| \|; Very preterm \| 1 \| 0.4 \| \| \|; Moderate preterm \| 13 \| 5.5 \| \| \|; Full term \| 221 \| 93.6 \| \| \| *Note. Extremely preterm = less than 28 weeks; Very preterm = 28 to 32 weeks; Moderate preterm = 32 to 37 weeks; Full term = equal to or more than 37 weeks.* |
| Furthermore, please explain whether the tools used and administered were validated in the population of the study or similar sample, this will help to draw stronger conclusions from the results. If so, add the cites. | Thank you for your valuable comment. The PCL-5, used to assess post-traumatic stress symptoms, has a validated Spanish adaptation (Blevins et al., 2015). Although it was originally developed for assessing trauma-related symptoms in the general population, it has been specifically validated for evaluating traumatic childbirth experiences (Arora et al., 2023). Furthermore, several studies have utilized this tool to assess the onset of post-traumatic stress symptoms related to childbirth (DeVita et al., 2023; Orovou et al., 2022; Vega-Sanz et al., 2024), instructing participants to complete the questionnaire with their childbirth experience in mind.  The social support questionnaire, Multidimensional Scale of Perceived Social Support (Zimet et al., 1988), has a Spanish adaptation (Landeta & Calvete, 2002), but it has not been specifically validated for use in the postpartum population. We have included it as such and provide the corresponding citation below.  Additionally, the questionnaire assessing childbirth conditions was specifically developed and validated for Spanish-speaking populations. It has been used for its intended purpose—to evaluate childbirth experiences, making it a suitable instrument for this study.  We have incorporated this clarification in the Instruments section of the Methodology, ensuring that the validation and appropriateness of the tools for this population are explicitly stated.  *“P-PTSS. The Post-traumatic Stress Symptom Checklist (PCL-5, 40) comprises 20 items and a Likert-type response scale (5 response options). This scale has a validated Spanish adaptation and also evidence of its cross-cultural validation (41). Although it was originally developed for assessing trauma-related symptoms in the general population, it has been specifically validated for evaluating traumatic childbirth experiences (42). It was specified that mothers should complete this questionnaire based on their childbirth experience.”*  *“Perceived social support.* *Multidimensional Scale of Perceived Social Support (MSPSS, 43). The MSPSS scale assesses the person's perceived social support at the global level, also consists of three subscales: perceived social support from family, from friends, and from a significant third party. It comprises 12 items (4 for each subscale) and a Likert-type response scale (7 response options). The present scale has been validated in Spanish (44) and present some evidence of cross-cultural validation (45) although it could be improved; however, it has not been specifically validated with a postpartum population.”* |
| In this part of the manuscript, my bigger concern flaws; that the authors used the Questionnaire on Birth Conditions. This questionnaire allows for the identification of some indicators of OV during childbirth (3,6,16,18); at the same time, it comprises 13 items. But who/how/when/where is supported that by selecting just these four items we could determine the presence of OV? If we consider the rule created by the authors "the items have been used as 13 independent indicators" including the procedures described and undertaken without information given; pretty much everything that the woman suffers, or even happens to her could be classified as OV (as an example an EMCS with GA ending with FFP or RBC transfusion, will possibly fall into this category; may the mum not remember the explanation given prior to the procedure and still a normal intervention in an emergency situation; thus this would not be appropriate). And I think this is the major problem of the study. I would recommend the authors to check this thoroughly. While all my other comments can be classified as minor adjustments, this is a major issue of the paper. | Thank you very much for your comment. First of all, we would like to clarify that for the analysis, all 13 indicators of the questionnaire were used—not only four. The numbers (3, 6, 16, 18) refer to the bibliographic references that support the notion that the content of these 13 indicators can be considered OV.  We totally agree that there could be cases where an unforeseen, highly severe situation requires immediate medical intervention, in which providing enough information to the woman about the procedure would not be possible. To avoid overrepresenting this item, we decided to add a question to the original 13 items specifying the type of procedure performed without providing information. This is detailed in the instrument’s description. Nevertheless, your comment has allowed us to add a clarification in the description. It now reads as follows:  *“Additionally, and to further clarify the information regarding the "procedures without information" item, exposure to the following procedures performed without providing information was assessed: vaginal exams; refusal to provide food; artificial rupture of membranes (AROM); episiotomy; instruction to always lie down during childbirth; intravenous catheter insertion; genital shaving; enema; abdominal compression (Kristeller maneuver).”*  In this way, we can ensure that the procedures without information reported in our paper refer specifically to these procedures—and not, for example, to the administration of emergency general anesthesia. |
| Results: This seems clearly approached and well presented. I can't see any manipulation from tables and every test seems appropriate. However, I´m missing a paragraph where all the main findings are presented at the beggining of the section. Just to make clear what to add to the current knowledge. | Thank you for your valuable feedback. We have incorporated an introductory paragraph at the beginning of the results section to emphasize the main contributions of the study. Below, we provide the revised version:  *“Our study provides valuable insights into the relationship between obstetric violence (OV) and perinatal post-traumatic stress symptoms (P-PTSS), identifying specific childbirth experiences that contribute to the development of P-PTSS. We identified three key aspects of the birth experience that serve as predictors of P-PTSS: feeling disqualified or mocked by healthcare staff during labor, lack of information about medical procedures, and being made to feel guilty by health professionals about a negative childbirth outcome. Furthermore, we highlight two protective factors: skin-to-skin contact in the immediate postpartum period, and perceived social support from family and friends”.* |
| Discussion: Seems properly addressed. But I would recommend to rewrite more cautiously on page 6, lines 11-24. Given the limitations of the study, I would rather be more wary of making assumptions. The manuscript is not well numbered, so I refer to page 26. | We sincerely appreciate your valuable comments and recommendations. We have carefully revised the discussion section and have reworded the conclusions to adopt a more cautious tone, considering the study's limitations. Specifically, we have avoided making definitive causal claims and have emphasized the need for further research to confirm and expand our findings. |
| The references must be checked as there are some mistakes and duplicate citations. E.g.: 3,15,16,19, 23, etc. | Thank you very much for your contribution. We have reviewed the references to ensure that there are no duplicates and have also corrected any formatting errors in the citations. |
| **2º Reviewer(s)' Comments to Author** |  |
| In all manuscripts, it is necessary to include most recent bibliographical references. | Thank you very much for your suggestion. We have reviewed the manuscript and updated the references accordingly. |
| Include information on how the participants were recruited, where and how the questionnaire was administered. | We greatly appreciate your contribution. We have completed the first paragraph of the Participants section, specifying how the dissemination was carried out, how the sample was obtained, and the way the participants completed the questionnaires. We attach below the revised paragraph:  *“A non-discriminatory exponential chain demonstration (i.e., snowball sampling technique) was performed to recruit the sample (N=236). The dissemination of the questionnaires was carried out through social networks, and participants were recruited via these platforms. They completed the questionnaires online through a Microsoft Forms survey. Sample recruitment began on 7 February 2023 and ended on 5 May 2023. The inclusion criteria for the study were being a woman over 25 years of age, residing in Spain, and being between the fourth and sixth week postpartum.”* |
| Include information and bibliographical references on the method section of the cross-cultural validation studies of the follow instruments: Questionnaire on Birth Conditions, PCL-5, Multidimensional Scale of Perceived Social Support for the Spanish population. | Thank you very much for your comment. Regarding the *Questionnaire on Birth Conditions,* as it is relatively recent, there is currently no published evidence regarding its cross-cultural validity. It is important to note that it was originally developed by Spanish researchers and has been used exclusively with a Spanish sample. Regarding PCL-5 and Multidimensional Scale of Perceived Social Support we have added references that provide information on its cross-cultural validation.  *“P-PTSS. The Post-traumatic Stress Symptom Checklist (PCL-5, 40) comprises 20 items and a Likert-type response scale (5 response options). This scale has a validated Spanish adaptation and also evidence of its cross-cultural validation (41). Although it was originally developed for assessing trauma-related symptoms in the general population, it has been specifically validated for evaluating traumatic childbirth experiences (42). It was specified that mothers should complete this questionnaire based on their childbirth experience.”*  *“Perceived social support.* *Multidimensional Scale of Perceived Social Support (MSPSS, 43). The MSPSS scale assesses the person's perceived social support at the global level, also consists of three subscales: perceived social support from family, from friends, and from a significant third party. It comprises 12 items (4 for each subscale) and a Likert-type response scale (7 response options). The present scale has been validated in Spanish (44) and present some evidence of cross-cultural validation (45) although it could be improved; however, it has not been specifically validated with a postpartum population.”* |
| The note in table 2: ‘The Birth Conditions questionnaire items were administered in Spanish as designed by Ramos and Avila. The research team performed the English translation of these items for the publication of this paper’ should be inserted and better described in the method, in the part of the instrument to which it refers. The authors should describe how this process was carried out. | Thank you very much for your comment. Indeed, the translation of these items was performed solely to enhance comprehension in the publication of this paper, and they are not empirically validated in English. At the bottom of Table 1, where it stated *“The research team performed the English translation of these items for the publication of this paper,”* we have now added *“They are not validated in the English version.”* Additionally, in the instrument description, we have added:  *“The questionnaire was used in its original Spanish version for the research, and the research team performed the English translation of these items to facilitate better understanding for this paper’s publication. The items were translated by bilingual colleagues from the research team.”*  Finally, we have added the original version of each item in the Table 1, to clarify as much as possible the information provided. |
| All the statistical analyses employed require the dependent variable (PTSD) to be parametric (normal distribution). Using parametric statistical analyses on non-parametric data could compromise the validity of the results. Therefore, the authors need to include in the method how the normality of the dependent variable was tested and what the results were. | Thank you for your suggestion. We have now included an explanation in the Data analysis section of Method apart regarding how the normality of the dependent variable (P-PTSS) was tested. We conducted the Shapiro-Wilk test, assessed skewness and kurtosis values, and visually inspected histograms and Q-Q plots. The results indicate that the data follow an approximately normal distribution, supporting the use of parametric analyses. Below, we provide the revised paragraph:  *“The normality of the dependent variable (P-PTSS) was assessed using the Shapiro-Wilk test (W = 0.98, p = 0.07), indicating no significant deviation from normality. Additionally, visual inspection of histograms and Q-Q plots showed an approximately normal distribution. The skewness (-0.45) and kurtosis (0.32) values fell within the acceptable range for normality (-1 to +1). These results confirm that P-PTSS follows an approximately normal distribution, supporting the use of parametric statistical analyses”.* |
| The authors need to explain in more detail how the linear regression models were carried out; the way it is written is confusing and does not make it possible to understand the modelling process. | We greatly appreciate your contribution. We have rewritten the explanation of the procedure for conducting the linear regression model to clarify it. Below, we provide the revised paragraph:  *“A linear regression analysis was conducted to explore the association between risk and protective factors and the dependent variable (P-PTSS), assessed between the fourth and sixth week postpartum. The model was built in three sequential steps to evaluate the incremental contribution of different sets of predictors: Step 1: Pregnancy-related factors – This step included medical complications during pregnancy as a predictor variable; Step 2: Childbirth-related factors – In this step, additional predictors were incorporated, including the type of childbirth (categorical variable), the presence of medical complications during delivery (dichotomous variable), indicators of obstetric violence (13 dichotomous variables), and the presence of skin-to-skin contact after birth (dichotomous variable); Step 3: Postpartum social support factors – The final step introduced perceived social support from friends, family, and a significant other (three continuous variables).*  *Each step allowed us to assess the incremental variance explained by each set of variables. The adjusted R² coefficient was used to evaluate the explanatory power of the model, and standardized beta coefficients (β) were reported to determine the strength and direction of the association between each predictor and P-PTSS. The final model results are presented in Table 4.”* |
| The authors should include in the method the level of statistical significance adopted. | Thank you for your suggestion. We have now specified the level of statistical significance adopted in our analysis within the Data Analysis section of the Methodology.  We include below the revised paragraph:  *“Statistical analyses were performed using IBM SPSS Statistics version 22 software. The significance level of p < 0.05 was considered statistically significant, while p < 0.01 was used to indicate stronger evidence of association. Initially, descriptive analyses of socio-demographic data and psychological measures of the participants were performed.”* |
| The authors should include in the method which regression parameters were used and how they were interpreted: e.g., to assess the association between independent and dependent variables, which regression parameter was used? To assess the explanatory power of the model, which parameter was used? | Thank you very much for your suggestion. We have included information on the statistical parameters used and their interpretation in the regression model within the Data Analysis section of the Methodology. Below, we provide the revised paragraph after these modifications:  *“Finally, to explore the association between risk and protective factors and the dependent variable (P-PTSS), a three-step linear regression analysis was conducted. The model examined the contribution of pregnancy-related factors (Step 1), childbirth-related factors (Step 2), and perceived social support (Step 3), allowing for an exploratory assessment of their potential influence on P-PTSS.* *To assess the association between each independent variable and the dependent variable (P-PTSS), we used the standardized beta coefficient (β) from the regression model. A positive β value indicates that higher levels of the independent variable are associated with higher P-PTSS scores, whereas a negative β value suggests an inverse relationship. Statistical significance was determined using p-values, with thresholds set at p < 0.05 and p < 0.01. To evaluate the explanatory power of the model, we used the adjusted R² coefficient, which represents the proportion of variance in P-PTSS explained by the independent variables while accounting for the number of predictors included in the model.”* |
| All the variables used in the linear regression model must be better described in the method (how they were obtained, how they were measured). | Thank you very much for your comment. We have added more information at the beginning of the procedure section in the methodology regarding the sample recruitment process and the evaluation procedure. Below, we provide the revised paragraph:  *“****Participants and Procedure:*** *A non-discriminatory exponential chain demonstration (i.e., snowball sampling technique) was performed to recruit the sample (N=236). The dissemination of the questionnaires was carried out through social networks, and participants were recruited via these platforms. They completed the questionnaires online through a Microsoft Forms survey. Sample recruitment began on 7 February 2023 and ended on 5 May 2023. The inclusion criteria for the study were being a woman over 25 years of age, residing in Spain, and being between the fourth and sixth week postpartum.”*  Additionally, we have reviewed the description of the instruments to ensure that the name of the variable being measured is specified, along with the type of scoring used in these analyses (continuous or dichotomous). We have also added information regarding the questions on sociodemographic data and pregnancy and childbirth details that were later included in the regression analysis. Below, we provide the revised paragraph:  ***“Measures:*** *Data was collected through online evaluation surveys using Microsoft Forms. The surveys included questions on socio-demographic and general aspects of pregnancy (such as medical complications during pregnancy) and childbirth (type of delivery, medical complications during childbirth, and whether skin-to-skin contact occurred after birth). The following questionnaires were used to assess risk factors for P-PTSS. “* |
| The authors should define whether they are building an explanatory (risk/protective factors) or predictive model (predictive factors), there is a difference between them and throughout the manuscript the authors use the two definitions (explanation and prediction) as synonyms. | We appreciate your clarification. Indeed, the model we are proposing is exploratory, as we do not yet know which specific elements of obstetric violence may influence the subsequent development of P-PTSS. We have thoroughly reviewed the manuscript to ensure consistency in terminology, referring exclusively to risk and protective factors rather than predictive factors. |
| In table 4 all VO variables should be described in full and not just item 1, 2, 3... | Thank you for your comment. We have added the description of each item. |
| page 4 ‘showed significant positive predictive power.’ What do the authors mean by this? Based on which statistics? This interpretation is confusing. | We sincerely appreciate your thorough review of this point. We have revised the paragraph to explicitly clarify the meaning of 'significant predictive power' by directly linking it to the β coefficients and p-values, and by reformulating the results in precise statistical terms.  Below, we present the revised paragraph for the interpretation of Table 4:  “*As shown in Table 4, the model accounted for 49.9% of the variance in P-PTSS between the fourth and sixth weeks postpartum. On the one hand, medical complications during pregnancy (β = 0.03, p = 0.62) did not show a statistically significant effect, indicating that these complications alone do not predict P-PTSS. On the other hand, childbirth-related variables explained 41.5% of the variance in P-PTSS. Specifically, three indicators of obstetric violence were significant predictors of higher P-PTSS scores: feeling disqualified or mocked by healthcare personnel during labour (β = 0.15, p = 0.02); lack of information about medical procedures (β = 0.12, p = 0.03); being made to feel guilty about negative childbirth outcomes by healthcare staff (β = 0.17, p = 0.004). These findings suggest that perceived mistreatment during childbirth is strongly associated with increased postpartum traumatic stress symptoms. Finally, postpartum factors explained an additional 8.4% of the variance in P-PTSS, with two variables emerging as significant protective factors: skin-to-skin contact was negatively associated with P-PTSS (β = -0.17, p = 0.008), indicating that immediate physical bonding with the newborn may help reduce posttraumatic stress symptoms; and perceived social support from friends and family also showed a negative association with P-PTSS (β = -0.26, p < 0.001 and β = -0.17, p = 0.02, respectively), suggesting that a supportive social environment plays a key role in mitigating postpartum psychological distress.”* |
| page 4 ‘Our results show that more than 10% of women in our sample suffered significantly from one or two types of childbirth-related posttraumatic symptoms, and 17% were close to clinical levels of posttraumatic childbirth-related symptomatology.’ These results should be described in the results section. | Thank you very much for your contribution. We have reviewed the text and further specified the participants' results on the PCL-5 at the beginning of the Instruments section. The revised text is as follows:  *“At four to six weeks postpartum, 17% of women met the criteria for a clinical level of P-PTSS, as they endorsed the minimum required number of items in each of the four symptom clusters (B to E), according to the scoring interpretation described in the Instruments section regarding the PCL-5 scale. Additionally, 10% of the sample exhibited a subclinical level of P-PTSS, meeting the item requirements in three out of the four clusters. However, at this point, the six-month duration criterion for a formal P-PTSS diagnosis had not yet been met. The majority of women, representing 74% of the sample, did not exhibit symptomatology consistent with P-PTSS.”*  Similarly, the reference to these results in the discussion focuses on analyzing the prevalence obtained in comparison with similar studies. Below, we provide the corresponding paragraph:  *“Our results indicate that 17% of women in our sample met the criteria for a clinical level of P-PTSS, and 10% exhibited a subclinical level of P-PTSS.These results are comparable to the prevalence of PTSD in the general population (47) during the COVID-19 pandemic (48), and higher than prevalences found in other studies (49, 50). This speaks to the fact that, despite the existence of most women who seem to undergo good experiences of childbirth, there is a significant proportion of mothers who are partially traumatized by their experience of giving birth. This may negatively affect their mental health, their transition to parenthood and, in the mid to long term, their children’s well-being. “* |
| Page 4 ‘clinical levels of posttraumatic childbirth-related symptomatology.’ It was not clear in the method how the authors made this classification. | Thank you very much for your suggestion. We have revised the wording in the Instruments section of the Methodology to clarify how the PCL-5 is scored and interpreted in the absence of defined cutoff points. Additionally, we have explained how this classification was used to categorize participants in our sample as having either a subclinical or clinical level of P-PTSS.  Below, we provide the final revised explanation:  *"Since no defined cutoff point exists, the authors suggest classifying the questionnaire items according to the symptom clusters established in the DSM-5. The items were grouped into the following clusters: B (items 1-5), C (items 6-7), D (items 8-14), and E (items 15-20). Each participant’s responses were then evaluated to determine how many items they endorsed within each cluster, following the DSM-5 diagnostic rule, which requires at least 1 item from cluster B, 1 from cluster C, 2 from cluster D, and 2 from cluster E to meet PTSD criteria.*  *Participants who met this criterion were categorized as having a clinical level of P-PTSS, even though the temporal criterion of a minimum symptom duration of six months for a formal P-PTSS diagnosis had not yet been met. Those who endorsed symptoms in three out of the four symptom clusters were classified as having a subclinical level of P-PTSS."* |
| The authors use risk factors as a synonym for predictive factors. There is a difference between these two types of variables, the authors should define which one it is. | Thank you very much for your review. We have specified that the regression model used is exploratory in nature so the word ‘predictive’ has been removed throughout the manuscript. |
| The last paragraph of the conclusion is not based on the methods and results of the study, I suggest rewriting. | Thank you for your thoughtful feedback. In response to your comment regarding the last paragraph of the conclusion, we have revised it to better align with the methods and results of our study. Specifically, we have introduced a statement connecting our findings with recent research emphasizing the importance of emotional care for mothers during childbirth, as well as the well-being of healthcare professionals. Additionally, we have refined the discussion to ensure it remains grounded in the scope of our study while acknowledging the need for further research on these issues.  *“Our results are aligned with a series of recent studies that highlight the importance of emotional care for mothers during childbirth and their first contact with their baby, as well as the need to protect the emotional well-being of healthcare professionals so they can provide adequate support to mothers during this vulnerable and significant moment.”* |
